# Hippocampal Dendritic Spines Modifications Induced by Perinatal Asphyxia

**DOI:** 10.1155/2012/873532

**Published:** 2012-05-07

**Authors:** G. E. Saraceno, R. Castilla, G. E. Barreto, J. Gonzalez, R. A. Kölliker-Frers, F. Capani

**Affiliations:** ^1^Laboratorio de Citoarquitectura y Plasticidad Neuronal, Instituto de Investigaciones Cardiológicas “Prof. Dr. Alberto C. Taquini” (ININCA), UBA-CONICET, C1122AAJ Buenos Aires, Argentina; ^2^Departamento de Nutrición y Bioquímica, Facultad de Ciencias, Pontificia Universidad Javeriana, Bogotá, Colombia

## Abstract

Perinatal asphyxia (PA) affects the synaptic function and morphological organization. In previous works, we have shown neuronal and synaptic changes in rat neostriatum subjected to hypoxia leading to long-term ubi-protein accumulation. Since F-actin is highly concentrated in dendritic spines, modifications in its organization could be related with alterations induced by hypoxia in the central nervous system (CNS). In the present study, we investigate the effects of PA on the actin cytoskeleton of hippocampal postsynaptic densities (PSD) in 4-month-old rats. PSD showed an increment in their thickness and in the level of ubiquitination. Correlative fluorescence-electron microscopy photooxidation showed a decrease in the number of F-actin-stained spines in hippocampal excitatory synapses subjected to PA. Although Western Blot analysis also showed a slight decrease in **β**-actin in PSD in PA animals, the difference was not significant. Taken together, this data suggests that long-term actin cytoskeleton might have role in PSD alterations which would be a spread phenomenon induced by PA.

## 1. Introduction

Dendritic spines are small protrusions that serve as a postsynaptic site for the 90% of the excitatory synapses in the CNS. Different kinds of dendritic spines were described based on their shape and their actin content in adult rat brains. Mushroom dendritic spines have stalks with a clear head differentiation, stubby spines are thick and have no neck, and thin spines are characterized as long and without neck [1]. Mushroom dendritic spines have a rich actin cytoskeleton network [1, 2], which is highly regulated by proteins that either stabilize the actin monomer (G-actin), such as thymosin or profiling, arp2/3, Rho-GTPase kinase contractin, or prevent polymerization and convert several polymers into small fragments of actin such as cofilin and gelsolin [3].

Several functions have been suggested for dendritic spines as they have been implicated in the mechanism of synaptic plasticity, learning and memory [4–7], and protein translocation [8]. Functional decline of dendritic spines is a consequence of synaptic loss in neurodegenerative disease and brain insults [9]. Furthermore, processes such as loss of dendritic spines, dendritic pruning, and loss of synaptic proteins precede neuronal death in many neurodegenerative disorders [10–12]. Moreover, activation and impaired function of the ubiquitin-proteasome pathway is thought to contribute to a number of neurodegenerative disorders [13]. Therefore, spine pathologies may be involved in different brain insults including hypoxia ischemia [14–17]. Perinatal asphyxia (PA) is a serious clinical complication with high mortality and morbidity [18]. Following PA, approximately 45% of newborn die and 25% have permanent neurological deficits including cerebral palsy, mental retardation and developmental delay, learning disabilities, visual and hearing problems, and different issues in school readiness [19–24].

In previous works, we have observed several alterations in striatum and hippocampal areas after PA, such as high level of ubiquitinization in dendritic spines, reactive gliosis, alterations in dendritic microtubular organization, and modification in cytoskeleton organization [17, 25, 26]. Given that numerous reports support the idea that dendritic spines are the main site damaged during brain ischemia [25, 27], we aimed to investigate whether dendritic spine changes are a spread feature induced by PA. For this purpose, we studied dendritic spine modifications in the *Stratum radiatum *of CA1 hippocampal area.

## 2. Material and Methods

### 2.1. Animals

 All procedures involving animals were approved by the Institutional Animal Care and Use Committee at the University of Buenos Aires (School of Medicine) and conducted according to the principles of the Guide for the Care and Use of Laboratory Animals (NIH Publications no. 80–23, revised 1996). Sprague-Dawley female rats in the fifteenth day of pregnancy were placed in individual cages and maintained on a 12 : 12 h light/dark cycle in a controlled temperature (21 ± 2°C) and humidity (65 ± 5%) environment. The animals had access to food (Purina chow) and tap water* ad libitum*. One group of animals (*n* = 10) was used as surrogate mothers, another group (*n* = 10) was assigned to PA procedures.

### 2.2. Materials

Eosin-phalloidin and Phalloidin-Alexa^568^ were purchased from Invitrogen (Carlsbad, CA). Secondary antibodies against mouse were obtained from Jackson ImmunoResearch Laboratories (West Grove, PA). Paraformaldehyde, EM grade glutaraldehyde, sodium cacodylate, and Durcopan ACM resin were obtained from Electron Microscopy Sciences (Fort Washington, PA); special welled tissue culture plates were obtained from MatTek (Ashland, MA). *β*-actin antibody was purchased from Sigma-Aldrich (cat no. A5441).

### 2.3. Induction of Asphyxia

Ten full-term pregnant rats on gestational day 22 were anesthetized [28], rapidly decapitated, and the uterus horns were isolated through an abdominal incision and placed in a water bath at 37°C for 19 min (subsevere PA: *n* = 10 full-term pregnant rats) [25, 26, 29–31]. We have used 19 min as the maximum time of PA because more than 20 minutes result in a survival rate lower than 3% [25]. Following asphyxia, the uterus horns were rapidly opened, the pups were removed, the amniotic fluid was cleaned, and the pups were stimulated to breathe by performing tactile stimulation with pieces of medical wipes for a few minutes until regular breathing was established. The umbilical cords were ligated, and the animals were left to recover for 1 hour under a heating lamp. When their physiological conditions improved, they were given to surrogate mothers who had delivered normally within the past 24 hours. The different groups of pups were marked and mixed with the surrogate mothers' normal litters (control animals (CTL) that were left undisturbed). We maintained litters of 10 pups with each surrogate mother.

### 2.4. Post-Asphyctic Procedure

 Four-month old male rats (6 per group) were used. Briefly, an intracardiac perfusion was performed with normal rat Ringer's at 35°C followed by fixative under deep anaesthesia (containing 50 mg/kg ketamine, 1 mg/kg rhompun and 5 mg/kg acetopromazine in sterile saline). For light microscopy analysis, rats were perfused with 4% formaldehyde (freshly made from paraformaldehyde) in 0.1 M phosphate buffer, pH 7.2. The brain was removed and fixed for 2 additional hours in the same solution at 4°C. Then, sections were embedded in Durcupan ACM resin. After removing the brain from the skull, it was postfixed in the same fixative during 2 h. Coronal or sagital sections (50–80 *μ*m) were made with a Vibratome (Leica). Some of these sections were stained with cresyl violet according to the procedures described in Capani et al. [32].

### 2.5. Photooxidation

Vibratome sections were washed with 50 mM glycine-PBS containing 0.5% cold water fish gelatin to block nonspecific binding. Following 30 min of washing, the sections were incubated on a shaker, in a solution of 0.05% of eosin phalloidin-0.5% cold-water fish gelatin/50 mM glycine-PBS for 2 h at 4°C. For light microscopic studies, phalloidin conjugated to Alexa^488^ was also used because of its superior fluorescent quantum yield compared to eosin. As a negative control, eosin-phalloidin was omitted. Tissue sections stained with eosin-phalloidin were mounted on glass-welled tissue culture dishes (Mat Tek Corp) pretreated with polyethylenimine. The slices were fixed again for 2–5 min with 2% glutaraldehyde in 0.1 M cacodylate buffer, rinsed in buffer for several minutes, and placed in 50 mM glycine and potassium cyanide in cacodylate buffer for an additional 5 min to reduce nonspecific staining. Photooxidation was performed on the Zeiss Axiovert described above, equipped with a 75-W xenon arc light source. The samples were immersed in a solution of 2.8 mM diaminobenzidine in 0.1 M sodium cacodylate at 4°C bubbled with pure O_2_, final pH 7.4, and then irradiated under conventional epifluorescence using a xenon lamp. After 6–8 min, a brownish reaction product began to appear in place of the fluorescence. The process was stopped by halting the excitation [1].

### 2.6. Electron Microscopy Procedure

Following photooxidation, tissue sections were rinsed in 0.1 M sodium cacodylate several times and incubated for 30 min with 1% osmium tetroxide in 0.1 M sodium cacodylate, pH 7.2. After several washes in double-distilled H_2_O, the sections were dehydrated in an ascending ethanol series, flat-embedded in Durcopan ACM resin, and polymerized for 24 h at 60°C. Serial thin sections (80–100 nm) were cut with Reichert Ultracut E ultramicrotome using glass knives and examined using a JEOL 100CX electron microscope at 80–100 keV. One set of thin sections was poststained with a combination of uranyl acetate and lead citrate. For E-PTA staining, sections were dehydrated in an ascending series of ethanol to 100% and stained for 1 h with 1% PTA stained prepared by dissolving 0.1 g of PTA in 10 mL of 100% ethanol and adding four drops of 95% ethanol [33]. Then, sections were embedded in Durcupan ACM resin.

### 2.7. Morphometric Analysis of Confocal Data

The volume fraction of immunoreactive material for phalloidin was estimated using the point-counting method of Weibel [34] and a grid delimiting 5000 *μ*m^2^ in the hippocampus. A total area of 75,000 *μ*m^2^ was evaluated in each animal. Percentage of reactive area was estimated using Image J Program (Image J 1.41^0^, NIH, USA). For electron microscopy analysis sampling, procedures were adapted from Harris et al. [35] and Capani et al. [1]. For analysis, spines were sampled from *Stratum radiatum* CA1 hippocampal area. All of the synapses that have the characteristic of mushrooms dendritic spines (head larger than the neck) were used in this study since mushrooms dendritic spines are the unique F-actin positive spines [1]. Random fields of neuropil containing at least one synapse were photographed at 10000x magnification and analyzed at a total magnification of 30000x. We analyzed 643 control spines and 638 spines for tissue subjected to PA.

### 2.8. Quantitative Analyses of E-PTA Material

CA1 Hippocampal specimens were selected for quantitative analyses based on the quality of E-PTA staining and the degree of ultrastructural preservation, as determined from conventionally stained material from the same animals. Samples were analyzed from controls (*n* = 4) and 19 min PA animals (*n* = 8). Tissue sections were cut at thickness of 100 nm and examined and photographed at 80 keV at a magnification of 8300x with a Zeiss 109 electron microscope. For each animal, five micrographs were obtained from hippocampus. As described above, each negative was digitized into a PC computer. Using NIH Image 1.6, PSDs were first manually outlined, and then the maximal thickness, minimum thickness, length, and total area of each PSD were determined. All synapses in which the postsynaptic density, intracleft line, and presynaptic grid were clearly visible were chosen for analysis. The selection criterion resulted in the analysis of between 30 and 50 PSDs per animal for each hippocampus.

### 2.9. Quantitative Analysis of Dendritic Spines

For analysis, spines were sampled from hippocampus. All of the synapses that have the characteristic of mushrooms type dendritic spines (head larger than the neck) were used in this study since mushrooms spines are the unique F-actin positive spines [1]. Random fields of neuropil containing at least one synapse were photographed at 10000x magnification and analyzed at a total magnification of 30000x. We analyzed 643 control spines and 638 spines for tissue subjected to hypoxia.

### 2.10. Subcellular Fractionation and Preparation of PSDs

Biochemical fractionation was performed as described previously by Saraceno et al. using the whole dorsal hippocampus [17] (CTL, *n* = 6; PA *n* = 6). Dounce homogenates (H) of the pellets in ice cold TEVP buffer (10 mM Tris-HCl, pH 7.4, 5 mM NaF, 1 mM Na3VO4, 1 mM EDTA, and 1 mM EGTA, 1.25 *μ*g/mL pepstatin A, 10 *μ*g/mL leupeptin, 2.5 *μ*g/mL aproptionin, 0.5 mM PMSF) containing 320 mM sucrose were centrifuged at 1000 × g to remove nuclei and large debris (P1). The supernatant (S1) was centrifuged at 10.000 × g for 10 min to obtain a crude synaptosomal fraction (P2) and subsequently was lysed hypoosmotically and centrifuged at 45.000 × g for 90 min to obtain a pellet of the synaptosomal membrane fraction (LP1). After each centrifugation, the resulting pellet was rinsed briefly with ice cold TEVP buffer before subsequent fractioning to avoid possible crossover contamination. Protein concentration was estimated by Bradford technique.

### 2.11. Western Blot

Western Blot analysis was carried out using LP1 fractions separated on 10% SDS-PAGE. Samples containing 50 *μ*g of protein from each group were applied to each lane. After electrophoresis, proteins were transferred to polyvinylidene difluoride (PVDF) membrane as described previously [36–38]. The membranes were incubated with a primary antibody anti-*β*-actin (Sigma, 1 : 1000) overnight at 4°C. Then, after appropriate washing procedures, they were incubated with horseradish peroxidase-conjugated anti-mouse secondary antibody for 2 hours at room temperature. The blots were developed with an ECL detection kit (Amersham). The films were scanned, and the optical density of protein bands was quantified using Gel Pro Analyzer software 3.1.00.00 (Media Cybernetics, USA). We used glyceraldehyde-3-phosphate dehydrogenase (GAPDH) as load controls [17, 39–41].

### 2.12. Statistical Analysis

The results were expressed as the means ± SEM. Student's *t*-test were carried out. A probability was considered to be significant at 5% or less. Statistical analyses were performed using the GraphPad Prism 5.03 for windows statistical package (GraphPad software, Inc, San Diego, CA, USA).

## 3. Results and Discussion

### 3.1. Microscopic Analysis of Hippocampal Sections

**Figure 1:**
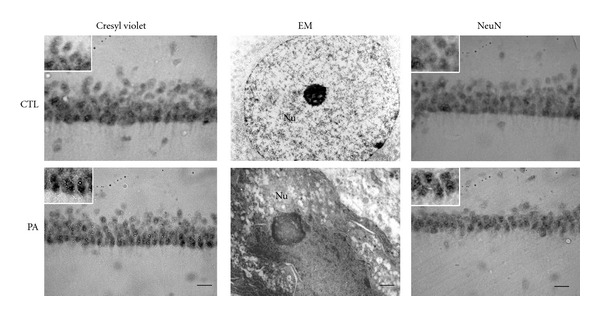
Micrographs of *Stratum radiatum* of CA1 hippocampal area from 4-month-old control rats and rats subjected to 19 min of PA. Vibratome sections of 50 *μ*m were cut and stained with cresyl violet (*Left*), analysed by electron microscopy (EM) (*Middle*) and NeuN immunostaining (*Right*). A clear nuclear condensation was observed after 19 min of PA. Electron micrograph showed that most of the condensed cells correspond to neurons in degeneration. Abnormal NeuN+ nuclei were increased in asphyctic animals respect to control group. Scale bar: 30 *μ*m and 0.5 *μ*m for EM. Nu: nucleus.

The study of nuclear morphology by Cresyl violet staining showed that PA animals present clear nuclear condensation 4 months after injury respect to CTL animals in the *Stratum radiatum* CA1 hippocampal area sections (Figure 1). Statistical analyses showed alterations in pyramidal neurons of hippocampal CA1 area, showing an abundance of pyknotic nuclei in asphyctic animals as compared to control animals (Table 1). Then, we employed neuronal nuclei (NeuN) immunolabeling to determine the nature of the cells presenting condensed nuclei (Figure 1). Statistical analyses showed no significant difference in the number of NeuN+ nuclei in the CA1 hippocampal area of asphyctic animals respect to controls. When we analyzed the cellular distribution of NeuN labeling, it was determinate that asphyctic animals showed a significant increase in the number of abnormal NeuN+ nuclei and a significant decrease in the number of normal NeuN+ nuclei in the CA1 hippocampal area compared with CTL group (Table 1). To determine the morphology of these cells, we performed a conventional electron microscopy study. Morphological analyses showed that most cells presenting condensed nuclei evidence dark cytoplasm with rare vacuoles and compaction, a hypertrophic nucleolus, a nucleus with a festoon shape, and a twisted nuclear envelope, corresponding to neurons in degeneration [25, 32, 42, 43] (Figure 1).

### 3.2. Modification in Hippocampal PSD Stained with E-PTA

**Figure 2:**
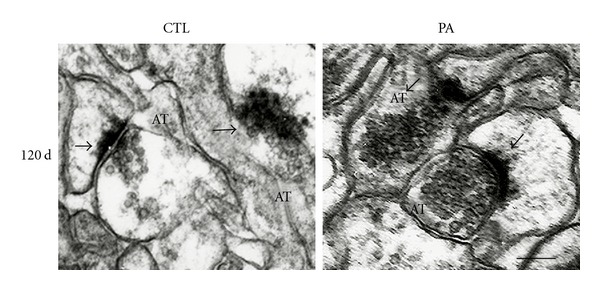
Electron micrographs of osmium-uranium-lead-stained synapses in *Stratum radiatum* of CA1 hippocampal area from 4-month-old control rats and rats subjected to PA. The synapses (arrows) were intact, and no obvious alterations were seen in these osmium-uranium-lead-stained synapses after PA. AT: axon terminal. Scale bar: 0.5 *μ*m.

**Figure 3:**
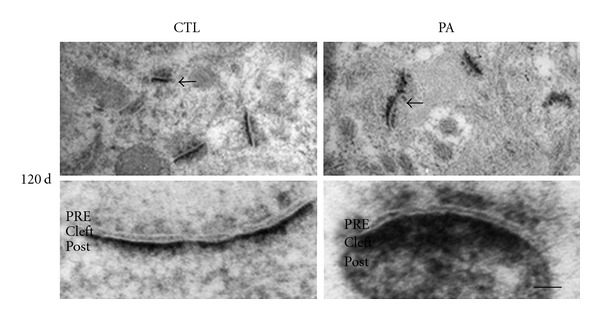
Electron micrographs of E-PTA-stained PSDs (arrows) in *Stratum radiatum* of CA1 hippocampal tissue section from 4-month-old control rats and rats subjected to 19 min of PA. Note the increased thickness and dispersed appearance of the PSDs in the asphyctic brains, compared with the control. Scale bar: 0.5 *μ*m. PRE: presynapses; PSD: postsynaptic density.

Osmium-lead-citrated staining showed no obvious alterations in the *Stratum radiatum* CA1 hippocampal area sections from 4-month-old CTL and PA rats (Figure 2). Presynaptic terminals, presynaptic vesicles, and ultrastructural organization of PSD were intact (Figure 2). On the other hand, E-PTA immunostaining showed clear alterations in synapses of rats subjected to PA (Figure 3). Following PA, the thickness of PSD increased as compared to controls (Figure 3). There was also a general increment in the amount of E-PTA-stained material in PSD of PA animals compared to controls. The statistical analysis performed confirmed these changes. *t* Student analyses for the area and length of the PSD and for the minimum and maximum thickness of the PSD were significant (*P* < 0.05). Post hoc tests revealed that the means of PSD area was significantly bigger as compared to the CTL group (*P* < 0.01) (Table 2). These inconsistencies between the osmium and E-PTA staining may be attributable to the fact that general heavy metal staining obscures the synaptic modifications occurring in post asphyctic tissue. In addition, it is possible that E-PTA stains different components in the PSD than osmium-uranium-lead methods. It has been known that PSDs stained with E-PTA are shorter and probably wider than those stained with the osmium-heavy metal method [44]. E-PTA preferentially stains protein(s) rich in basic amino acid residues, including lysine, arginine, and histidine, such as collagen or histones [45]. In contrast, conventional heavy metal staining stains a wide type of lipids and cytoskeletal and cytoplasmic elements [44]. Since both markers stain different components, this might explain why E-PTA staining is more effective to detect the PSDs alterations than heavy metal-stained sections.

Consistent with other studies in different models of ischemia [27, 33, 43] and using this long-term PA model [1], we did not observe any alterations in the subcellular organization of hippocampus material stained with osmium-heavy metals. However, we observed a marked increase in E-PTA-stained material in subsevere PA. Although not too much data is available about the mechanism of cell death during PA [25, 30], these findings suggest that the increased in the thickness could be related with the degradation of abnormal proteins probably before neurons trigger death mechanisms. Thus, we hypothesize that some early signals triggered in PSDs could induce late neuronal alterations in post asphyctic hippocampal tissue.

### 3.3. Ubiquitin-Protein Conjugates in Hippocampal PSDs

**Figure 4:**
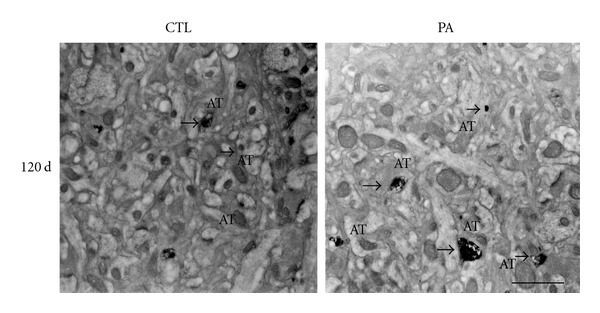
Electron micrographs of ubiquitin immunolabeling from *Stratum radiatum* of CA1 hippocampal tissue of 4-month-old control rats and rats subjected 19 min of PA. Strong ubiquitin stained was observed in asphyctic PSDs (arrowheads). In control animals ubiquitin staining was very weak and rare. AT: axon terminal. Scale bar: 0.5 *μ*m.

Since we and others demonstrated that E-PTA-stained aggregates could be composed of abnormal protein [25, 33, 46], we performed immunoelectron microscopy following the procedures previously described by Capani et al. [25] in order to detect ubiquitin (Figure 4). We observed ubiquitinated synaptic proteins after 19 min of PA in the *Stratum radiatum* CA1 hippocampal area sections, while negative controls, in which the primary antibody was omitted, did not show immunolabeling (data not shown). We rarely observed ubiquitin labeling in PSD of CTL animals. Taking these results into account, we could suggest that aggregates of ubi-proteins are present in PSDs of asphyctic animals, as it was observed in some neurodegenerative diseases [47].

Even though data about cell death mechanisms during PA are scarce [25, 30], these findings suggest that PSD thickening could be related to degradation of abnormal proteins, probably before cell death mechanisms are triggered in neurons. Consistent with this view, persistent ubiquitination was found in the PSD of hippocampal neurons [46, 48] after transient cerebral ischemia [43], which suggests that increased ubi-protein conjugates might produce protein damage. In addition, damage in protein can be produced by the increment in ROS production and calpain activation as consequence of a rise in Ca levels after hypoxic-ischemic insult [1, 49, 50]. On the other hand, while other heat shock proteins reversibly attach to denatured proteins and help to refold or reassemble them, ubiquitin-conjugated proteins are degraded by 26 S proteasome [47]. PA insult activates the ubiquitin pathway, which might affect neuronal survival by damaged protein accumulation. Since neurons do not have the capacity to remove it, ubi-protein accumulation leads neurons to death.

### 3.4. F-Actin Changes in Hippocampal Dendritic Spines Induced by PA

**Figure 5:**
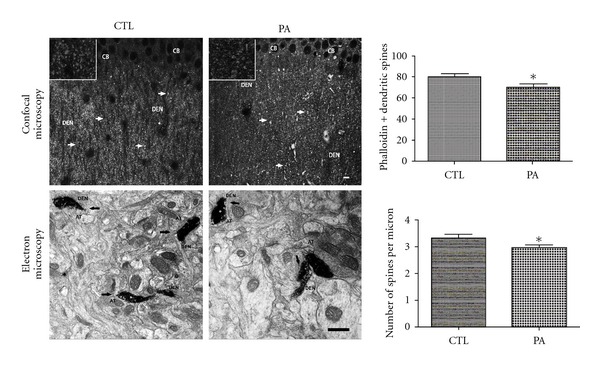
*Top*: confocal microscope images of Phalloidin-Alexa^568^ from *Stratum radiatum* of CA1 hippocampal tissue from 4-month-old control and asphyctic rats. A decrease in the punctate staining was observed after 19 minutes of PA (arrows). The assessment of the percentage of the reactive area from *Stratum radiatum* of CA1 hippocampal Phalloidin-Alexa^568^ staining in PA rats showed a decrement in the reactivity area staining with phalloidin. *P < 0.01. Bars and error bars represent mean + SEM. DEN: dendrites; CB: cell body. Scale bar: 10 *μ*m. *Bottom*: electron micrograph of photooxidated area in the *Stratum radiatum* of CA1 hippocampal tissue of 4-month-old rats. Arrows point out the dendritic spines stained. A decrement in the number of the F-actin-positive spines was observed after 19 min of PA. AT: axon terminal; DEN: dendritic shaft. Scale bar: 1 *μ*m. The graph shows the assessment of the number of spines per field from *Stratum radiatum* of CA1 hippocampal slices. A significant decrement in the number of positive F-actin spines was observed in the PA group in comparison with the CTL group. *P < 0.01. Bars and error bars represent mean + SEM.

Brain hypoxia-ischemia triggers an early increment of glutamate in the extracellular space at synaptic level [51]. High levels of glutamate produce a cascade of events in dendritic spines that lead to cell death [25, 31, 33, 43, 48, 52–55]. Since structure and function of dendritic spines are dynamically regulated by different cellular pathways acting on the actin cytoskeleton, we used light and electron microscopic techniques that had previously been used in our laboratory [1, 56] and others [4, 8] to study F-actin modifications induced by PA. By confocal microscope analyses, we observed dendritic spines represented by punctate staining using Phalloidin-Alexa^568^. PA animals showed a decrease in punctate staining respect to CTL group (Figure 5 *Top*) (*P* < 0.01). The morphometric analysis confirmed these data. Since F-actin is mostly concentrated in mushroom dendritic spines [1], this decrement is tightly related with the F-actin contained in the dendritic spines.

**Figure 6:**
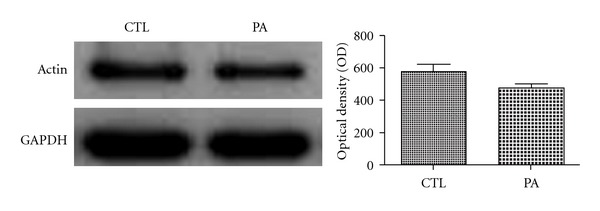
Immunoblots of hippocampal sinaptosomal fractions of 4-month-old CTL and PA rats. We used the glyceraldehyde-3-phosphate dehydrogenase (GAPDH) as loading controls. The assessment of the percentage of optical density of immunoblots from the 4-month-old CTL and PA rats showed no significant difference in the optical density with respect to control group (CTL). *P* = 0,058. Bars and error bars represent mean + SEM.

Electron microscopic analyses of spine population in the photooxidated samples confirmed confocal microscopic observations. When we analyzed different dendritic spine populations, we observed that only the number of mushroom dendritic spines, the only F-actin-positive spines in control animals, showed a significant decrease after 19 min of PA (*P* < 0.05) (Figure 5 *Bottom*). In contrast, synapses did not show any sign of evident degeneration in asphyxic rats. Isolated synaptosome (LP1) fractions were analyzed by immunoblotting using anti-*β*-actin antibody and quantified (Figures 5 and 6). Statistical analysis showed no significant differences in mean optical densities of F-actin bands (*P* = n.s.) from PA and control group. However, PA animals showed a decrease in amount of *β*-actin with respect to CTL animals (*P* = 0,058). Both *in vivo* and *in vitro* studies showed high concentration of *β*-actin in dendritic spines are involved in the organization of the synapses in adult brains [57–60]. Although we observed a reduction of the number of spines F-actin positive in PA animals, the maintenance of *β*-actin concentration observed in synaptosomal fraction may represent the cytoskeletal support of a stable dendritic spines structure that maintains, thus, the potential morphological plasticity in circumstances where adaptive changes in synaptic connectivity are adequate [60]. Consistent with this point of view, disruption of receptor-scaffold proteins as NMDAR-PSD 95, which depends on actin polymerization interactions, can prevent cell death after ischemia [61].

Actin cytoskeleton is highly regulated by several actin binding proteins (ABP) [3]. Many ABPs have been involved in the regulation of neuronal death during ischemia. Changes in spine morphology are strongly linked with some ABP such as gelsolin. Several studies using different models of neuronal cell death have demonstrated that endogenous gelsolin's antiapoptotic properties correlated to its dynamic actions in the cytoskeleton. Gelsolin-null neurons have higher rates of cell death and a rapid and sustained elevation of Ca^2+^ levels following glucose/oxygen deprivation, as well as augmented cytosolic Ca^2+^ levels in nerve terminals following *in vitro* depolarization [52]. Gelsolin also diminishes infarct size after ischemia, preventing neuronal death [62]. Furthermore, the increment in histone acetylation induces upregulation of gelsolin, dramatically reducing the levels of actin filaments and cell death following cerebral ischemia in mice [63]. Although a recent study by Gisselsson et al. [15] has shown that actin depolymerization prevents neuronal death, we hypothesize that the decrease in *β*-actin in synaptosomal fraction could also be related with cell death observed after PA insult, as this process is connected with an abnormal ubi-protein increment.

In addition, our group has previously observed learning; reference and working spatial memory impairments in the Morris water maze in 3-months-old rat, subjected to acute asphyxia immediately after birth, using the hypoxic-ischemia model described in the present manuscript [31]. It is well known that the performance in these spatial tests is disrupted after hippocampal damage [64]. Moreover, deficits were observed in the exploration of a novel environment. Therefore, synaptic modifications observed in asphyctic animals could be related with behavioral deficits previously described by our group [31].

## 4. Conclusions

These findings suggest that excessive protein ubiquitination in hippocampal PSD, 4 months after a subsevere PA insult, seems to be related to the increment in protein accumulation in this area. In spite of this increment, we observed a decrease in *β*-actin which suggests that PA is damaging the actin cytoskeleton. Moreover, the amount of *β*-actin in PA animals is correlated with the decrement in the number of mushroom-shaped dendritic spines. Although further studies will be necessary to determine the role of ubi-protein accumulation in PSD, we could speculate that PSD alterations might be involved in the generation of an aberrant biochemical pathway leading to long-term modifications in the brain of PA animals, as we described in a previous paper [25]. In agreement with this point of view, Alzheimer's disease has a deleterious action on the actin cytoskeleton linked with PSD, leading to dendritic spine dysfunction and synaptic degeneration [65].

## Figures and Tables

**Table 1:** Analysis of neuron cells in CA1 hippocampal area.

Groups	Neurons with pyknotic nuclei	Neurons NeuN+	Normal neurons	Abnormal neurons
CTL	2.94 ± 0.2	75.34 ± 2.4	72.20 ± 0.5	3.04 ± 0.5
PA	6.29 ± 1.3*	71.58 ± 5.5	64.12 ± 0.4*	7.46 ± 1.2*

Data are expressed as means ± SD. Significative differences were obtain using Student *t*-test. *P < 0.05.

**Table 2:** Analysis of PSDs features in CA1 hippocampal area.

Groups	Área × 10^3^ (nm^2^)	Length (nm)	Minimum thickness (nm)	Maximum thickness (nm)
CTL	2.0 ± 0.2	97.1 ± 2.4	16.2 ± 0.5	43.0 ± 2.5
PA	5.7 ± 1.3**	105.1 ± 5.5	35.6 ± 0.4**	80.1 ± 2.2**

Data are expressed as means ± SD. Significative differences were obtained using Student *t*-test. **P < 0.01.
